# Is smaller better? Vaccine targeting recombinant receptor-binding domain might hold the key for mass production of effective prophylactics to fight the COVID-19 pandemic

**DOI:** 10.1038/s41392-020-00317-1

**Published:** 2020-10-06

**Authors:** Manish Muhuri, Guangping Gao

**Affiliations:** 1 Horae Gene Therapy Center, University of Massachusetts Medical School, Worcester, MA USA; 2 Department of Microbiology and Physiological Systems, University of Massachusetts Medical School, Worcester, MA USA; 3 Li Weibo Institute for Rare Diseases Research, University of Massachusetts Medical School, Worcester, MA USA

**Keywords:** Vaccines, Infectious diseases, Adaptive immunity

A recent report by Yang et al. published in Nature reported a recombinant vaccine utilizing recombinant receptor-binding domain (RBD) of SARS-CoV-2 Spike Protein.^1^ This vaccine candidate successfully induced potent functional antibody responses in the immunized mice, rabbits, and non-human primates. The study highlights the critical role of the immunogenicity of the RBD domain upon SARS-CoV-2 infection and the alternate vaccine designs that could serve as effective prophylactics against the pandemic.

Coronavirus disease 2019 (COVID-19) is a type of viral pneumonia that is caused by severe acute respiratory syndrome–coronavirus 2 (SARS-CoV-2). The virus is highly transmissible between humans and has spread rapidly (estimated R_0_ = 5.7), causing a worldwide pandemic that has had a devastating impact on global health and the world economy. As of August 8, 2020, almost 20 million people worldwide have been infected with SARS-CoV-2 and more than 750,000 deaths have been reported.^2^ Along with SARS-CoV and Middle East respiratory syndrome–coronavirus (MERS-CoV), SARS-CoV-2 is the third coronavirus to cause severe respiratory illness in humans. Although several drugs are under investigation to treat the disease and some have entered clinical trials, future outbreaks by related coronaviruses originating from spill-over reservoirs are highly likely. Thus, there is an urgent need to rapidly develop and deploy safe and effective vaccines to immunize an extraordinarily large number of individuals in order to protect the entire global community from the continued threat of morbidity and mortality.

**Fig. 1:**
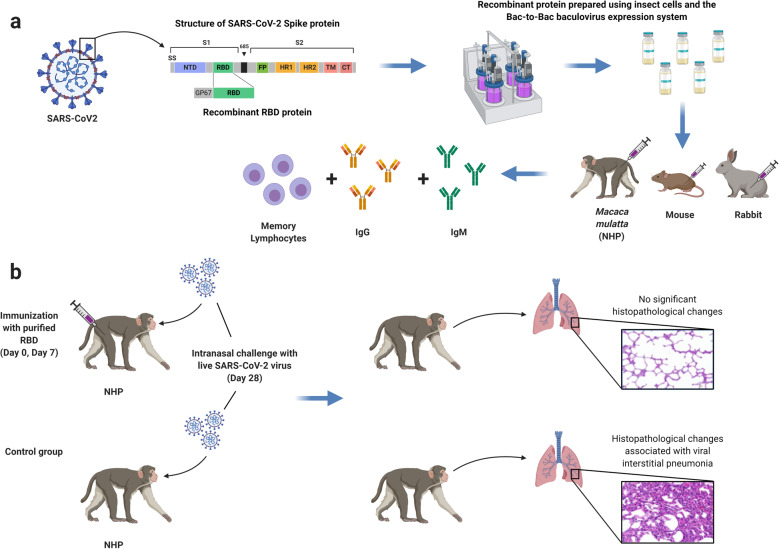
Design and production of an RBD-based vaccine and inspecting its protective effects in in vivo models. **a** The Spike protein of SARS-CoV-2 is known to be critical for virus’ entry into the cells. Rational design of the vaccine involves the insertion of a GP67 signal peptide upstream to the RBD domain of the spike protein. The recombinant protein is produced using insect cells and the Bac-to-Bac baculovirus expression system. The purified protein formulation is then injected via intramuscular injections in mice, rabbits, and non-human primates (NHPs; Macaca mulatta). The sera from the injected animals are immunophenotyped to profile the immune response against the RBD protein. IgG, IgM, and memory lymphocytes are consistently produced across all immunized animals. The indicated domains and elements of the spike protein, including signal sequence (SS), N-terminal domain (NTD), receptor-binding domain (RBD), heptad repeat1 and 2 (HR1 and HR2), transmembrane domain (TM), and cytoplasmic domain (CT), are marked. **b** NHPs are sequentially immunized on day 0 and day 7 intramuscularly with purified RBD or control treatment followed by intranasal challenge with live SARS-CoV-2 on day 28. Neutralization activity was measured from their sera, viral load was measured from throat, lung, and anal swabs, and histopathological changes in lung tissue were analyzed by light microscopy. Immunized NHPs displayed potent neutralization, minimal viral load with the appearance of normal lung tissue histopathology

Currently, the global COVID-19 vaccine R&D landscape includes more than 200 vaccine candidates, of which several have moved into clinical development.^3^ It has been structurally and functionally established that the S protein, specifically the receptor-binding domain (RBD), binds to the angiotensin-converting enzyme 2 (ACE2) receptor on the surface of human cells to promote host cell entry, making the viral spike an attractive therapeutic and vaccine target.^4^ At least 60 vaccine platforms that are in clinical trials involve injection of one of the recombinant SARS-CoV-2 proteins to immunize a healthy individual to trigger a protective immune response against future exposures; with a large majority utilizing the spike protein. In an alternate strategy, Yang et al. developed a rationally designed recombinant protein vaccine comprising residues 319–545 of the SARS-CoV-2 spike-protein RBD (Fig. 1).^1^ The recombinant protein was produced from insect cells using the Bac-to-Bac Baculovirus Expression System. Notably, proteins derived from the baculovirus expression system generally assume a correctly folded conformation and enable mass production in a relatively simple, rapid, and scalable manner making them commercially feasible. Structural insights into the purified RBD protein revealed several glycosylation sites, which were identified and mapped to ensure they may not interfere with ACE2 receptor recognition/binding.

Thereafter, the humoral response of the recombinant RBD, in combination with alum as an adjuvant, was assessed upon intramuscular injections in mice, rabbits, and non-human primates (NHPs). Anti-RBD-specific IgG and IgM were readily detectable in the sera of immunized mice as early as 7 days post-injection. This antibody response was dose dependent and also displayed potent neutralization activity both in rabbits and NHPs. Furthermore, the authors investigated the protective effects of immunization upon subsequent exposure to SARS-CoV-2. Vaccination with a single low dose of RBD in mice could effectively block RBD binding to ACE2 receptors in cells *in vitro* in the presence of sera from immunized mice. Similar results were seen in NHPs where sera from immunized *Macaca mulatta* 7 days post-immunization could significantly reduce SARS-CoV-2 pseudovirus infection in ACE2 expressing Huh7 cells. More importantly, two sequential intramuscular injections at day 0 and day 7, with either 20 μg or 40 μg of purified RBD seemed to credibly protect NHPs from subsequent infection by live SARS-CoV-2 when challenged intranasally 28 days after the first dose. Lung tissue, throat swabs, and anal swabs of the NHPs were assessed for viral genomic RNA and subgenomic RNA (sgRNA; indicative of viral replication) 3–7 days after virus exposure. Even though viral genomic RNA was detected in the throat and anal swabs (but not lung tissue), no sgRNA was detected in any tissue from the vaccinated groups indicating no evidence of viral replication and hence, efficacious protection.

Having established the efficacy of the vaccination strategy, it is critical to understand the mechanisms for pathogenesis and immune pathways that contribute to the humoral and cellular immune response induced by the vaccine. Such insights might prove essential for the development and improvement of effectual prophylactic and interventional strategies. The authors observed that vaccination of the mice deficient in Cd4^−/−^, Sting1^−/−^, Casp1^−/−^, Nlrp3^−/−^ IL-1β^−/−^, Tlr2^−/−^, and Tlr4^−/−^ (but not Cd8^−/−^) led to a reduction in the induction of RBD-specific IgG. However, RBD-vaccinated mice were also found to stimulate the production of memory T cells—CD4 + CD44^high^IL-4 + , CD4 + CD44^high^IFN-γ-, and CD8 + CD44^high^IFN-γ—that are known to play a vital role in mounting an antiviral response with subsequent exposures to the pathogen. To further elucidate the role of cellular response for this vaccination platform, adoptive transfer of immunized sera or splenic T cells from the vaccinated mice to naïve mice was performed. While splenic T cells did not protect against SARS-CoV-2, the transferred sera demonstrated a strong protective effect with no detectable viral replication in the recipient mice after live SARS-CoV-2 challenge.

Given the unusual choice of recombinant protein used in this study, the authors compared the neutralization potential of anti-RBD antibodies to the other domains of the spike protein—the extracellular domain protein (ECD), S1-subunit protein (S1) and S2-subunit protein (S2). They found that the rationally designed recombinant RBD vaccine had a much higher viral neutralization activity than other forms of the spike-protein tested. The safety profile of the vaccine was evaluated in NHPs (*n* = 50) and no adverse events and abnormality in clinical pathology, hematology, and histopathology were reported.

Taken together, this study highlights the importance of the RBD domain in S protein immunogenicity and the rationale for selecting it as a vaccine candidate. When the authors compared the sera from COVID-19 patients and healthy donors for their immunoglobulin profile, IgG and IgM levels against RBD were elevated in COVID-19 patients. Also, this strategy based on RBD has been reinforced by the recent findings that the majority of the human monoclonal antibodies and the neutralizing activity is directed against RBD in the sera of COVID-19 patients.^5^ Additionally, as many as six independent vaccine development initiatives are using RBD as the recombinant protein for immunization—one of which is already in clinical trials.^3^ All the findings from this well designed and well-executed study are very encouraging and strongly supportive of further clinical development of this vaccine candidate.
